# Insights into cell robustness against lignocellulosic inhibitors and insoluble solids in bioethanol production processes

**DOI:** 10.1038/s41598-021-04554-4

**Published:** 2022-01-11

**Authors:** Antonio D. Moreno, Cristina González-Fernández, Elia Tomás-Pejó

**Affiliations:** 1 Biotechnological Processes Unit, IMDEA Energy Institute, Avda. Ramón de la Sagra 3, 28935 Móstoles, Madrid, Spain; 2 Advanced Biofuels and Bioproducts Unit, CIEMAT, Avda. Complutense 40, 28040 Madrid, Spain

**Keywords:** Biotechnology, Microbiology

## Abstract

Increasing yeast robustness against lignocellulosic-derived inhibitors and insoluble solids in bioethanol production is essential for the transition to a bio-based economy. This work evaluates the effect exerted by insoluble solids on yeast tolerance to inhibitory compounds, which is crucial in high gravity processes. Adaptive laboratory evolution (ALE) was applied on a xylose-fermenting *Saccharomyces cerevisiae* strain to simultaneously increase the tolerance to lignocellulosic inhibitors and insoluble solids. The evolved strain gave rise to a fivefold increase in bioethanol yield in fermentation experiments with high concentration of inhibitors and 10% (w/v) of water insoluble solids. This strain also produced 5% (*P* > 0.01) more ethanol than the parental in simultaneous saccharification and fermentation of steam-exploded wheat straw, mainly due to an increased xylose consumption. In response to the stress conditions (solids and inhibitors) imposed in ALE, cells induced the expression of genes related to cell wall integrity (*SRL1*, *CWP2*, *WSC2* and *WSC4*) and general stress response (e.g., *CDC5*, *DUN1*, *CTT1*, *GRE1*), simultaneously repressing genes related to protein synthesis and iron transport and homeostasis (e.g., *FTR1*, *ARN1*, *FRE1*), ultimately leading to the improved phenotype. These results contribute towards understanding molecular mechanisms that cells might use to convert lignocellulosic substrates effectively.

## Introduction

Lignocellulose is present in agricultural residues such as rice straw, wheat straw, olive pruning, and gardening wastes. It is a renewable energy reservoir and a sustainable feedstock for chemicals and fuels. The efficient use of lignocellulosic resources will significantly boost the transition towards a bio-based economy. In this sense, the extensive research progress during the last decades has promoted the construction of several industrial-scale plants for lignocellulosic ethanol production^1^. Conversion of lignocellulose under high gravity conditions (i.e., high substrate concentrations) is crucial to achieve high ethanol titers and reduce distillation costs. However, high-gravity technology is still very challenging due to the increase in complexity of the corresponding medium (insoluble solids, inhibitors, etc.). These difficulties associated to the use of high substrate loadings negatively influence cell performance during the fermentation.

Biotechnological conversion of lignocellulose into bioethanol involves pretreatment, enzymatic hydrolysis, fermentation and product recovery as major steps. Pretreatment is required to alter the physicochemical properties of lignocellulosic biomass and ease the accessibility of the hydrolytic enzymes to carbohydrates. Most common pretreatment technologies lead, however, to biomass degradation and formation of several compounds, which may inhibit the subsequent saccharification and fermentation steps. In particular, the effects that these inhibitory compounds exert on yeast have been widely explored^2–5^, and many different studies have targeted the overcoming of such effects^6–9^.

Along with the inhibitors, insoluble solids are also present in the media during simultaneous saccharification and fermentation (SSF) and consolidated bioprocesses (CBP). These configuration strategies have been claimed as two promising options to produce lignocellulosic ethanol due to the costs saving potential resulting from the integration of the saccharification and fermentation steps. The integration of these stages reduces the required equipment, decreases the overall process length, and increases the fermentation efficiency^10,11^. However, the presence of insoluble solids may also influence the fermentation performance of yeast cells as well as yeasts tolerance to inhibitory compounds^12^, especially at the initial fermentation stages when the concentration of solids is high (solids concentration is diminished along the time due to enzymatic hydrolysis of carbohydrates). In the particular case of CBP processes, hydrolysis of cellulose usually exhibits low rates^13^, thus implying the presence of insoluble solids at high concentrations for longer periods than in SSF.

Solid insoluble particles produce shear stress, induce damage in brewing yeast, promote changes in gene expression and accumulation of intracellular reactive oxygen species^12,14^. Notwithstanding, the potential effects that insoluble solids have on bioethanol producing yeasts have been frequently underestimated. Several studies have demonstrated the tolerance of yeast cells towards lignocellulose-derived inhibitors during fermentation of liquid prehydrolysates while the same concentration of inhibitory products completely inhibited cells in SSF processes^15,16^. Thus, determining the impact of insoluble solids on yeasts is therefore crucial to identify future research lines for the development of more robust and efficient strains with potential applications at industrial scale.

The present work aims at evaluating the effect exerted by insoluble solids on the tolerance of yeast cells to inhibitory compounds, which is of great relevance in SSF/CBP processes at high gravity. For this purpose, the fermentation performance of the yeast *Saccharomyces cerevisiae* F12, a recombinant xylose-fermenting strain successfully used in SSF processes^8,10^, was investigated in presence and absence of lignocellulosic insoluble solids and/or inhibitors to determine its tolerance towards these stressors. Since, adaptive laboratory evolution (ALE) is effective for obtaining novel yeast strains better adapted to the challenging bioethanol production conditions^8,17–19^, *S. cerevisiae* F12 was subjected to an ALE procedure in the presence of both lignocellulosic degradation compounds and insoluble solids. Subsequently, the genetic changes for facing such challenging environment were identified. In evolutionary procedures, cells are forced to replicate under certain restricting conditions during long periods of time. The modulation of the environment during evolution increases the rate of spontaneous mutagenesis and so, designing an appropriated evolution strategy is crucial for the success of the process.

Overall, this study reports for the first time the evolution of yeast cells on insoluble solids and inhibitors to better adapt them to high gravity technology. This work also reveals the most important variations in gene expression that take place during the evolution process. Results presented herein will pave the way for identifying new strategies to develop novel strains to be efficiently applied in high-gravity lignocellulosic conversion processes (i.e., with inhibitors and insoluble solids) at the industrial scale.

## Materials and methods

### Insoluble solids from steam-exploded wheat straw

The collection of the used wheat straw complied with relevant institutional, national, and international guidelines and legislation. Wheat straw was pretreated in a 10-L steam explosion reactor at 210 °C for 5 min. Slurry was separated into liquid fraction and water insoluble solid (WIS) fraction by vacuum filtration using a Büchner funnel. The resulting WIS fraction was thoroughly washed with distilled water to remove soluble inhibitory compounds and embedded sugars. The WIS fraction had the following composition in terms of % dry weight (w/w): 52.1 cellulose, 8.0 xylan, 0.2 arabinose and 33.9 lignin.

In order to assess the effect of solids on yeast fermentation, one portion of WIS was dried at 40 °C and added at 5% (w/v) and 10% (w/v) to the synthetic fermentation media depending on the experimental conditions. Both the whole slurry and the WIS fraction were used as substrate for SSF experiments.

### Inhibitor mix

The inhibitor mix was prepared by using commercial compounds to give a final composition equivalent to those commonly found in steam-exploded lignocellulosic hydrolysates (2.1 g/L furfural, 0.3 g/L 5-HMF, 13.4 g/L acetic acid, 10.5 g/L formic acid, 0.4 g/L ferulic acid, 0.2 g/L syringaldehyde, and 0.1 g/L vanillin)^20^. This inhibitor mix was used as selection pressure during the evolutionary engineering approach and in fermentation experiments at 50% (v/v), and 100% (v/v) dilution in presence and absence of WIS.

### Microorganism and cell propagation

Recombinant *S. cerevisiae* F12 was kindly supplied by Professor Lisbeth Olsson from Chalmers University of Technology (Sweden). This strain was genetically modified to consume xylose by overexpressing the endogenous gene encoding xylulokinase and by introducing genes encoding xylose reductase and xylitol dehydrogenase from *Scheffersomyces stipitis*^21^.

For preinoculum preparation, *S. cerevisiae* F12 cells were grown in 100-mL shake flasks with 20 mL YPD medium (10 g/L yeast extract, 20 g/L peptone, 20 g/L glucose) in an orbital shaker at 150 rpm and 32 °C for 16 h. Cells were harvested by centrifugation (3000 g, 8 min, 25 °C) and diluted with the corresponding medium to get the appropriate inoculum size.

### Adaptive laboratory evolution experiment

*S. cerevisiae* F12 was subjected to ALE to increase its robustness towards lignocellulose-derived inhibitors and insoluble solids. ALE was performed by sequential batch cultivation of yeast cells in 250-mL Erlenmeyer flasks containing 50 mL of the corresponding medium. Cells were incubated at 150 rpm, 32 °C and pH 5.0 with an initial OD_600_ of 0.1. YNB (Conda, Cat.1553.00) supplemented with 7.5 g/L (NH_4_)_2_ SO_4_ was used as basal medium. ALE experiment was distributed in different stages according to Table 1. 4-mm diameter glass beads (Hecht Karl™ 1401/4) were used as insoluble solids to progressively evolve cells and facilitate their subsequent recovery. The experiment started by adding 20% (w/w) insoluble solids to a medium containing glucose and xylose at a final concentration of 10 g/L each. The xylose:glucose ratio (w:w) increased gradually to 10:10, 15:5, and 18:2 as evolution proceeded. Simultaneously, solids were combined with increased concentrations of the inhibitory mix, starting from 12.5% (v/v) to 80% (v/v).

**Table 1:** Strategy followed during the ALE in terms of insoluble solids, xylose:glucose and inhibitors concentration for each round.

Round Nº	Solids % (w/v)	Xylose:Glucose (g/L)	Inhibitors % (v/v)	Nº of rounds*
**Round 0**	20	10:10	0	2
	20	15:5	12.5	7
	20	15:5	15	2
	20	15:5	17.5	4
	20	15:5	20	2
	20	15:5	22.5	5
	20	15:5	25	13
	20	15:5	27.5	11
	20	18:2	30	2
	20	18:2	50	8
	20	18:2	70	4
**Round 88**	20	18:2	80	4

*Number of rounds under each condition.

Selection of spontaneous mutants with improved tolerance was based on increased specific growth rates. When an improvement in the yeast growth was detected (measured as OD_600_ basis), xylose:glucose ratio and inhibitor concentration in the evolution media was increased (Table 1). Each round of evolution started by inoculating an aliquot of cells from the previous shake flask culture at a final OD_600_ of 0.1. The evolved strain was obtained after 88 rounds of evolutions (≈ 2.200 generations). For isolation of single colonies, cells from the final round were harvested, diluted accordingly, and grown for 36 h at 32 ºC in a YPXD-agar plate containing 10 g/L glucose, 10 g/L xylose and 20 g/L agar. One of the most prominent colonies was selected and named as evolved *S. cerevisiae* F12 strain.

### Fermentation tests

Synthetic fermentation media containing 10 g/L glucose, 10 g/L xylose, 2 g/L NH_4_Cl, 2 g/L KH_2_PO_4_, 0.3 g/L MgSO_4_·7 H_2_O, and 5 g/L yeast extract were used to assess the fermentation performance of *S. cerevisiae* F12 in presence of WIS and/or inhibitors under the conditions stated in Table 2. Fermentation tests were carried out in triplicate in sterilized 250-mL Erlenmeyer flasks with 100 mL medium at 150 rpm and 32 °C for 48 h with 1 g/L (dry weight) of inoculum concentration.

**Table 2:** Concentration of inhibitors and/or WIS during fermentation assays.

Assay	Inhibitor mix % (v/v)	WIS % (w/v)
CONTROL	0	0
I50	50	0
I100	100	0
WIS5	0	5
WIS10	0	10
I50_WIS5	50	5
I50_WIS10	50	10
I100_WIS5	100	5
I100_WIS10	100	10

In a first set of experiments, the influence of lignocellulosic degradation compounds on parental *S. cerevisiae* F12 was evaluated by using 50% and 100% (v/v) of the inhibitor mix. Subsequently, the effect exerted by solids on the fermentation performance of yeast cells was assessed by adding 5% and 10% of WIS (w/v). Finally, cells were subjected to fermentation in the presence of different combinations of solids (5% and 10% WIS (w/v)) and inhibitors (50% and 100% (v/v) of the inhibitor mix) to identify any potential synergism between these two stressors.

Evolved *S. cerevisiae* F12 strain was also used under the most sever conditions: i) the presence of 100% (v/v) inhibitor mix, ii) the presence of 10% of WIS (w/v) and, iii) the combination of both 100% (v/v) inhibitors and 10% of WIS (w/v).

### Simultaneous saccharification and fermentation assays

Parental and evolved *S. cerevisiae* F12 strains were used in SSF with steam-pretreated wheat straw at high substrate loadings to evaluate the success of the evolutionary engineering approach. For that, the whole slurry supplemented with nutrients (2 g/L NH_4_Cl, 2 g/L KH_2_PO_4_, 0.3 g/L MgSO_4_·7 H_2_O and 5 g/L yeast extract) was used at a final concentration of 20% total solids (TS) (w/v). Due to the highly inhibitory potential of the slurry, the WIS fraction was also subjected to SSF at 20% (w/v) substrate concentration and supplemented with the same nutrients. Since most of the xylose remained in the liquid fraction when collecting the WIS fraction, 30 g/L xylose were added to the SSF media to enrich the fraction of this sugar and mimic the sugar composition in the slurry.

All SSF experiments were run in triplicate at 150 rpm, 35 °C and pH 5.5 (NaOH 4 M) for 72 h in 250-mL Erlenmeyer flasks containing 100 mL of medium. SSF assays were supplemented with 15 FPU/g substrate of Celluclast 1.5 L (60 FPU/mL) 15 IU/g substrate of ß-glucosidase NS50010 (900 IU/mL) and 1 g/L (dry weight) of either parental or evolved *S. cerevisiae* F12.

### Analytical methods

The chemical composition of the WIS fraction was analyzed by using the standard methods for determination of structural carbohydrates and lignin in biomass (LAP-002, LAP- 003, and LAP-019) of the National Renewable Energy Laboratory (NREL). The full description for these methods can be found in the following link [https://www.nrel.gov/bioenergy/biomass-compositional-analysis.html].

Glucose, xylose, xylitol and ethanol were determined and quantified by high-performance liquid chromatography (HPLC) using an Agilent HPLC 1200 Series equipped with a refractive index detector and an Aminex HPX-87H Ion Exclusion column operating at 50 °C with 5 mM H_2_SO_4_ (0.6 mL/min) as elution buffer.

Statistics were performed to estimate the mean and standard deviation during fermentation and SSF assays. Analysis of variance (ANOVA) was used for comparison between assays using the software *Statgraphics Centurion XVIII*. The level of significance was set at *P* < 0.05, *P* < 0.01, and *P* < 0.001.

### Microarray analysis

Total RNA was extracted from the evolved and parental *S. cerevisiae* F12 after 4 h of fermentation in YPXD medium supplemented or not with 40% (w/w) insoluble solids and 100% (v/v) inhibitor mix. To avoid interferences with RNA extraction method, 4-mm diameter glass beads (Hecht Karl™ 1401/4) were used as insoluble solids instead of pretreated WIS. Cells (5 mL) were withdrawn, cooled on ice, centrifuged (4000 g, 2 min, 4 °C), frozen in liquid nitrogen and stored at −80 °C until further analysis. Trizol reagent (Invitrogen) was used for RNA isolation according to the manufacturer’s protocol. Samples were treated with RNase-free DNase I (Qiagen) to prevent DNA contamination. The concentration and purity of RNA was measured using an UV-light Omega spectrophotometer. Furthermore, RNA integrity was determined using the Bioanalyzer 2100 (Agilent) and only samples with 260/280 > 1.8, 260/230 > 2.0, and RNA Integrity Number (RIN) > 8.0 were subjected to further analysis.

After RNA isolation, samples were treated as explained previously^12^, using the GeneChip™ Yeast Genome 2.0 Array (Affymetrix®) to determine gene expression. Raw data were processed with RMA algorithm included in Affymetrix® Expression Console™ for normalization and gene level analysis. Three microarray experiments corresponding to three independent RNA replicates were processed and analyzed for each experimental condition. Fold changes between experimental conditions were calculated as a quotient between the mean of the gene expression signals. The LIMMA package included in Babelomics software package [http://www.babelomics.org] was used for statistical analysis^22^. Those values with a false discovery rates (FDR) < 0.05 were considered as significant. Genes with Log2-fold change > 1 or < (−1) were included for further analysis. Microarray experiments were also analyzed by Piano software [http://biomet-toolbox.chalmers.se]^23^. Differentially expressed genes were identified with an FDR < 0.05 selection cut-off and the corresponding heat map was simultaneously obtained.

Microarray data were submitted to the NCBI GEO with GSE159167 as accession number [https://www.ncbi.nlm.nih.gov/geo/query/acc.cgi?acc=GSE159167].

Differentially expressed genes were classified by YeastMine according to their main known/proposed functions^24^. In this context, both downregulated and upregulated genes were used to investigate and categorize them according to their biological processes and molecular functions by the gene ontology (GO)-annotations. Finally, network analysis of known/predicted protein–protein interactions was evaluated by STRING software v11^25^.

## Results and discussion

### Effect of WIS and/or inhibitors on yeast fermentation

**Figure 1:**
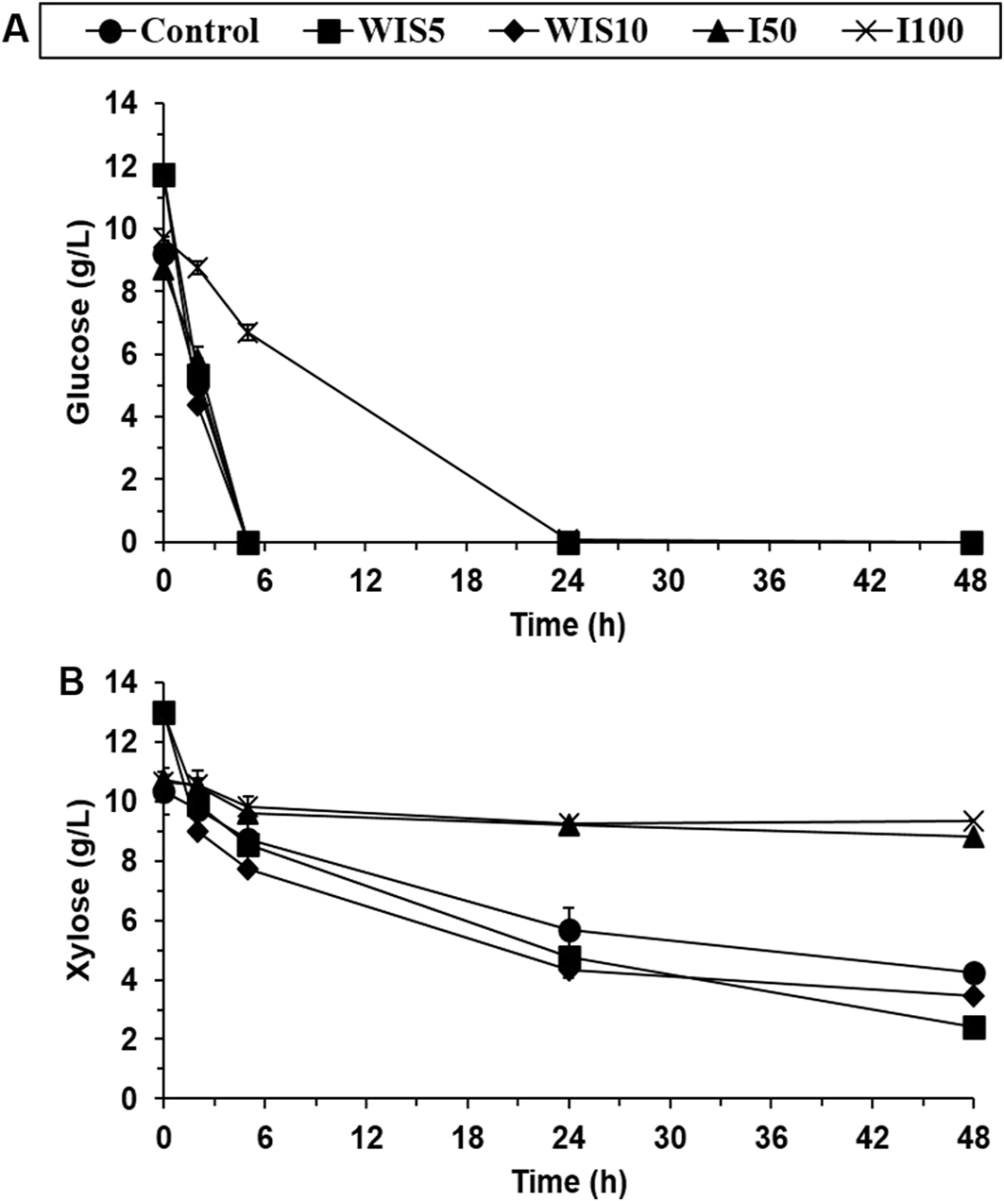
Time-course for (**A**) glucose and (**B**) xylose consumption during fermentation assays in presence of different concentrations of WIS and lignocellulose-derived inhibitors.

This study assessed how the presence of inhibitors and WIS may influence yeast fermentation under the conditions stated in Table 2. As shown in Fig. 1A, no differences were observed in terms of glucose consumption rates or residual glucose in fermentation experiments with 50% (v/v) inhibitor mix or 5–10% (w/v) of WIS when compared to control assays without insoluble solids and inhibitors. In these cases, no lag phase was detected and glucose was exhausted within the first 5 h of fermentation. This result agrees with Koppram and co-workers that showed no differences in the consumption of 20 g/L glucose when control fermentation (with no WIS in the medium) was compared to fermentations in the presence of 2, 5, 10, and 12% WIS (w/w)^26^. The presence of 100% (v/v) of inhibitor mix reduced, however, the glucose consumption rates, reaching glucose exhaustion at 24 h (Fig. 1A) and corroborating the well-known effect that high concentration of inhibitors exerts on yeast cells, which in turns hampers glucose utilization^27,28^.

In contrast to glucose conversion, the presence of lignocellulose-derived inhibitors exhibited a strong inhibition effect during the xylose conversion phase (Fig. 1B). In this case, the addition of 50% and 100% (v/v) of inhibitor mix resulted in restricted xylose assimilation by cells, which only consumed 18% and 12% of the initial xylose concentration, respectively (Table 3). The higher susceptibility of xylose fermentation to lignocellulose-derived inhibitors compared to that of glucose fermentation has already been shown in several studies^29,30^. Since xylose utilization has been proven to provide less energy in the form of ATP compared to glucose^31^, and response to inhibitors requires high energy levels, the presence of inhibitors may have a stronger effect on yeast when xylose is the utilized carbon source. Furthermore, it is likely that the genetic modifications needed to construct xylose-fermenting yeasts alter their cell metabolic homeostasis affecting the inhibitor tolerance^2^.

**Table 3:** Glucose and xylose consumption and ethanol yields during fermentation assays at different inhibitors and WIS concentrations.

Assay	Strain	Glucose consumption (%)	Xylose consumption (%)	Y_ETOH_ (g/g)
CONTROL	Parental *S. cerevisiae* F12	100 ± 0	60 ± 1	0.28 ± 0.01
I50		100 ± 0	18 ± 1	0.22 ± 0.00
I100		100 ± 0	12 ± 0	0.19 ± 0.04
WIS5		100 ± 0	82 ± 3	0.20 ± 0.00
WIS10		100 ± 0	74 ± 1	0.21 ± 0.01
I50_WIS5		100 ± 0	22 ± 6	0.22 ± 0.02
I50_WIS10		100 ± 0	22 ± 1	0.19 ± 0.01
I100_WIS5		77 ± 2	8 ± 3	0.16 ± 0.00
I100_WIS10		18 ± 4	0 ± 1	0.05 ± 0.01
I100	Evolved *S. cerevisiae* F12	100 ± 0	64 ± 1	0.25 ± 0.04
WIS10		100 ± 0	59 ± 1	0.24 ± 0.01
I100_WIS10		100 ± 0	21 ± 3	0.24 ± 0.01

By contrast, the presence of 5% (w/w) or 10% (w/w) WIS slightly increased xylose consumption when compared to control assays (Fig. 1B). Tricarboxylic acids (TCA) cycle was identified as one of the targets of transcriptional regulation to optimize xylose utilization. Thus, intensive TCA cycle was assigned to be important for xylose metabolism in xylose-recombinant *S. cerevisiae* strains^32^. In the same context, regulation of the stress response and amino acid metabolism have been shown as two important strategies for an effective xylose utilization in a recombinant xylose-fermenting *S. cerevisiae* strain^32,33^. Strikingly, Moreno and co-workers identified amino acids biosynthesis and carboxylic acid metabolic processes among the major overexpressed biological processes in *S. cerevisiae* F12 grown in glucose media with insoluble solids^12^. Thus, WIS may affect yeast cells by promoting xylose utilization when no other lignocellulose-derived inhibitor is present.

Despite the increase in xylose consumption, ethanol yields in presence of WIS were 0.20–0.21 g/g. This value was 25–30% lower than the obtained in control assays (0.28 g/g) (Table 3). Lower ethanol yields are commonly linked to an increase in xylitol production^34^. Nevertheless, similar xylitol concentrations (< 0.1 g/L) were found in control and fermentation assays with only WIS. Thus, slight differences in cell growth in presence of WIS or redistribution of metabolic fluxes to cope with the challenging conditions imposed by WIS may result in lower ethanol yields.

As mentioned before, Koppram and co-workers^26^ did not observed differences in ethanol yields when adding up to 12% (w/w) of WIS to fermentation media with 20 g/L glucose, reaching ethanol yields of 0.32 g/g. However, when adding 40% (w/w) and 60% (w/w) insoluble solids, Moreno and colleagues^12^ showed a decrease in ethanol yield in glucose media from 0.37 g/g without solids to 0.35 g/g and 0.22 g/g, respectively. It is worth mentioning that previous studies only utilized glucose as carbon source. In spite of promoting xylose consumption in presence of 5% (w/w) and 10% (w/w) of WIS, the reduced ethanol yields obtained in this study indicated that xylose fermentation was more prone to be affected by stressful conditions.

Lower ethanol yields than those obtained for control assays were also found when lignocellulosic inhibitors were present, reaching 0.22 g/g and 0.19 g/g with 50% (v/v) and 100% (v/v) of the inhibitor mix, respectively (Table 3). As previously commented, less than 20% of the initial xylose concentration was consumed by non-evolved yeast cells (Fig. 1B). In addition, when increasing the inhibitor content from 50% (v/v) to 100% (v/v), the glucose consumption rates decreased by threefold (from 1.8 g/L h to 0.6 g/L h) at the initial stages of the fermentation process (5 h) (Fig. 1A). This result is indicative of the high inhibitory potential of lignocellulose-derived inhibitors, especially during the xylose assimilation phase.

**Figure 2:**
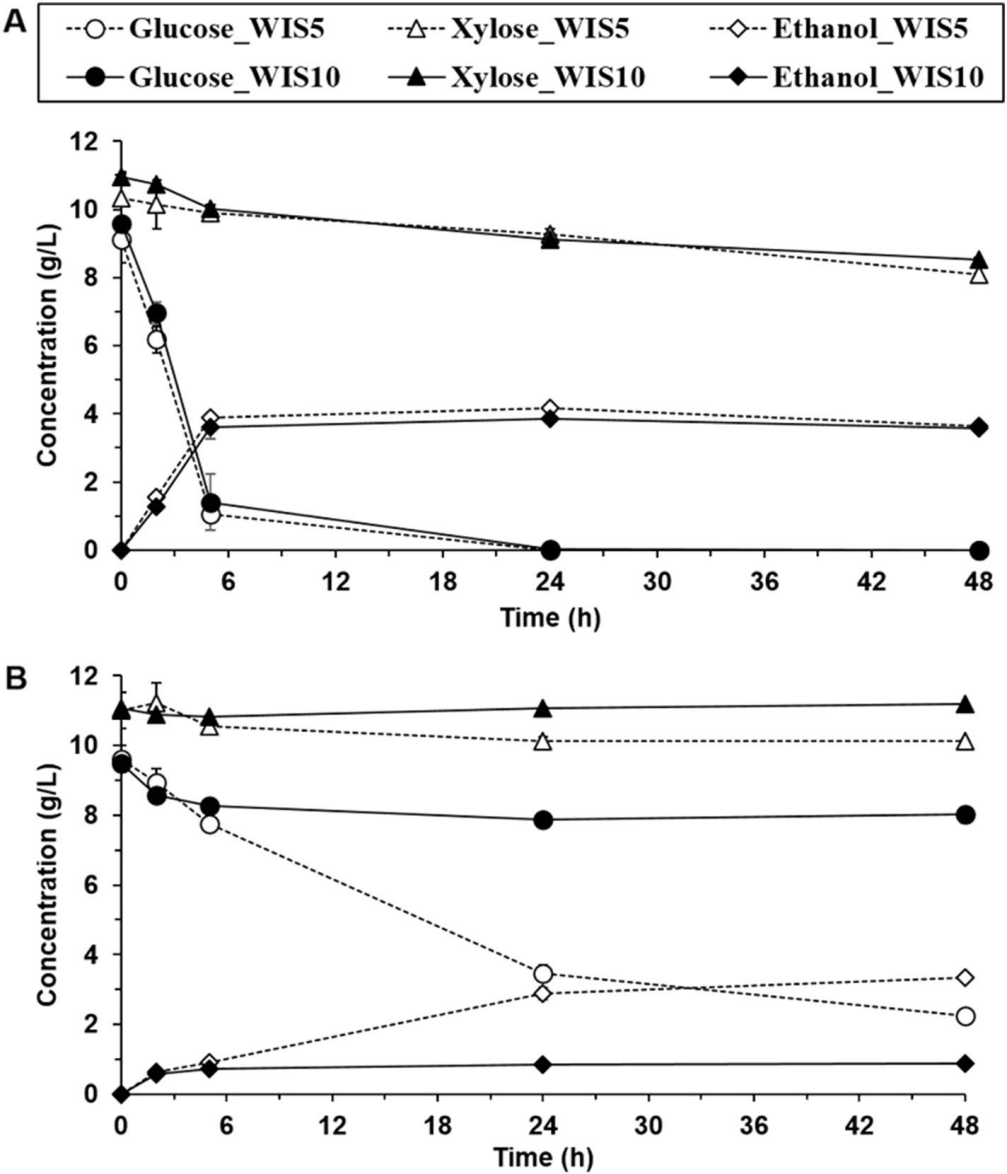
Fermentation assays with (**A**) 50% and (**B**) 100% (v/v) inhibitor mix in presence of 5% and 10% (w/w) WIS.

Besides the detrimental effect that the presence of WIS exhibited on ethanol yields in fermentation experiments with 10 g/L glucose and 10 g/L xylose, the influence that the presence of WIS has on the inhibitory tolerance of *S. cerevisiae* F12 was also studied. For such a goal, 50% (v/v) or 100% (v/v) inhibitor mix were combined with 5% (w/v) or 10% (w/v) of WIS in different fermentation tests. As it is shown in Fig. 2A, when using 50% (v/v) of inhibitor mix, glucose was exhausted within the first 24 h, and 22% of the xylose was consumed after 48 h of fermentation. In this case, the ethanol yield was 0.22 g/g and 0.19 g/g with 5% (w/v) and 10% (w/v) of WIS, respectively (Table 3). These ethanol yields were similar than those obtained when only 50% (v/v) of inhibitor mix was added (Table 3), indicating that yeast tolerance was not significantly affected by the presence of WIS at low inhibitor concentration. On the other hand, when 100% (v/v) of the inhibitor mix was combined with either 5% or 10% (w/v) of WIS neither glucose nor xylose were exhausted in 48-h long fermentation (Fig. 2B). Furthermore, marked differences were observed in ethanol yield in comparison with only 100% (v/v) of the inhibitor mix (Table 3). When 5% WIS (w/v) were added together with 100% (v/v) of the inhibitor mix, about 80% of the initial glucose and 10% of the initial xylose were consumed after 48 h of fermentation, reaching an ethanol yield of 0.16 g/g. However, 10% (w/v) of WIS together with 100% (v/v) inhibitor mix resulted in 80% less ethanol when compared to only 100% (v/v) inhibitor mix. The lower ethanol concentrations were directly linked to a completely hampered xylose consumption and to a limited glucose consumption. These results clearly showed a synergistic effect when combining both lignocellulose-derived inhibitors and WIS and pointed out to the presence of WIS as a crucial factor when yeast cells have to deal with high concentrations of inhibitory compounds.

In the present work, an increase in xylose uptake was observed when 50% (v/v) of inhibitor mix was combined with WIS compared with only 50% (v/v) inhibitors (Table 3). This result supported the hypothesis that the presence of insoluble solids may promote xylose consumption in absence of biomass degradations compounds or when inhibitors are present at low concentrations. In this sense, Koppram and co-workers^26^ studied the effect of steam-pretreated birch WIS on the glucose consumption and yeast tolerance to either HMF (1 g/L), furfural (1 g/L), syringaldehyde (0.8 g/L) or acetic acid (9 g/L). These authors reported higher glucose uptake rates when low concentrations of these compounds were simultaneously present with WIS compared to those obtained in the absence of solids^26^. In the same study, a proteomic analysis revealed up-regulation of glycolytic enzymes and ATP synthases in the presence of acetic acid and WIS, strongly indicating an increased generation of energy in the presence of both stressors (WIS and inhibitors) which could be the reason for the increased sugar consumption.

The ALE procedure in WIS-rich and inhibitor-rich media (Table 1) resulted in an evolved *S. cerevisiae* F12 with improved abilities to cope with the combination of both inhibitors and WIS. When compared with the parental strain, a decrease in the xylose consumption was observed when only WIS (10% w/v) was present in the fermentation broth (Table 3). However, in presence of 100% (v/v) inhibitor mix, xylose consumption increased from 12% with parental *S. cerevisiae* F12 to 64% with evolved cells which was also translated in an increase of ethanol yield from 0.19 g/g to 0.25 g/g. These results suggest that evolution procedure primarily favored changes to increased tolerance to inhibitors that could be detrimental to cope with the sole presence of insoluble solids. The success of ALE was evident when comparing parental and evolved *S. cerevisiae* F12 performance at the most challenging conditions (i.e. 100% (v/v) of inhibitor mix and 10% (w/v) of WIS). In this case, parental *S. cerevisiae* F12 did not consume any xylose and ethanol yield was as low as 0.05 g/g. On the other hand, xylose consumption and ethanol yield increased to 21% and 0.24 g/g, respectively, when using the evolved strain proving the effectiveness of ALE as strategy to increase tolerance to a combination of stressors.

### Simultaneous saccharification and fermentation at high substrate loading

**Figure 3:**
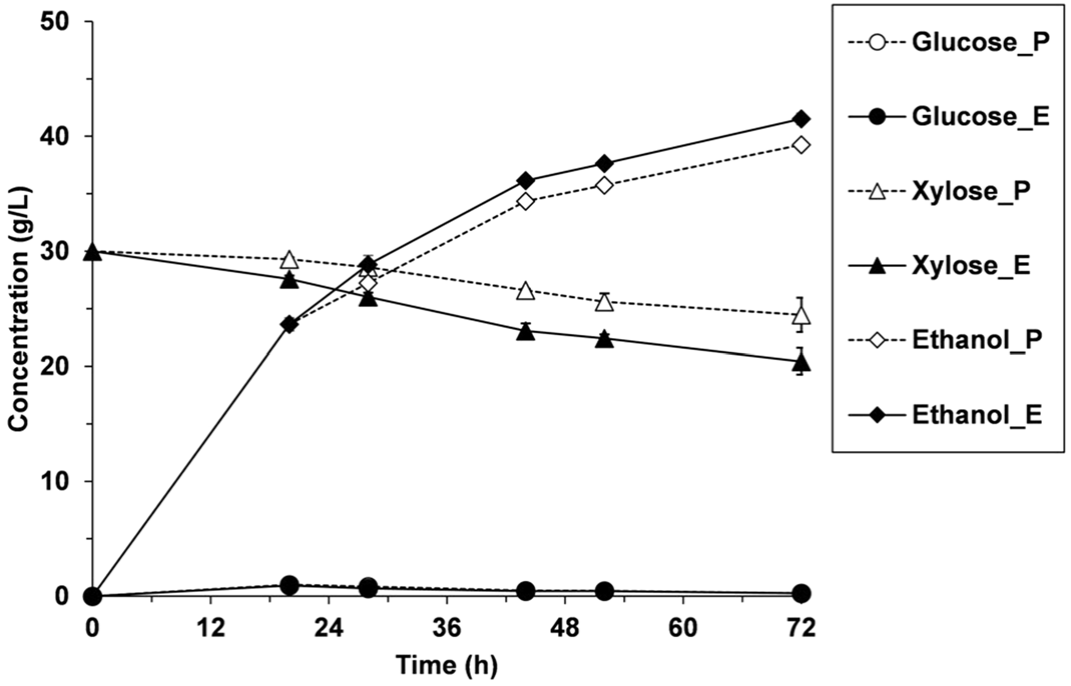
SSF of steam-exploded wheat straw (WIS supplemented with xylose), using the parental (P) and evolved (E) *S. cerevisiae* F12.

Parental *S. cerevisiae* F12 was used in SSF to evaluate its fermentation performance and cell robustness under high substrate loading. When using the whole slurry at a concentration of 20% TS (w/v), no ethanol was produced during SSF processes (data not shown). Although parental cells were able to cope with 100% (v/v) inhibitory mix in absence of WIS (Fig. 1), the presence of solids and inhibitors in SSF of slurry led to complete cell inhibition. This fact pointed to a reduced tolerance to inhibitors in presence of high solids content. In this case, the progressive liquefaction of the solids during the first hours of SSF was not sufficient to overcome the effect that WIS had on yeast tolerance to inhibitors. Nevertheless, when using 20% WIS (w/v) supplemented with xylose (i.e. absence of inhibitors), parental *S. cerevisiae* F12 was capable of fermenting both glucose and xylose, reaching a maximum ethanol concentration of 39.3 ± 0.4 g/L (Fig. 3).

In SSF from WIS, *S. cerevisiae* F12 assimilated glucose immediately upon enzymatic hydrolysis, thus maintaining a low glucose concentration during the fermentation process (Fig. 3). In contrast, limited xylose consumption was shown within 72 h of SSF. Recombinant *S. cerevisiae* cells use the same transport systems to incorporate both glucose and xylose inside the yeast cell^35,36^. The uptake of xylose through the transport system has been reported to have significantly lower affinities for xylose than for glucose^37^. In this sense, the xylose uptake is strongly inhibited when glucose is present. This fact is decisive in mixed sugar fermentations with recombinant *S. cerevisiae* strains because this yeast does not utilize xylose unless glucose is significantly depleted. In this case, glucose concentration was below 0.5 g/L during SSF process, and the limited xylose consumption could be therefore explained due to the stressful fermentation conditions.

The robustness of the evolved strain was evaluated under the same SSF conditions than the parental strain. Similar to the parental *S. cerevisiae* F12, the evolved strain was totally inhibited during SSF processes of the whole slurry at 20% TS (w/v) (data not shown). However, in the SSF from WIS, the evolved strain produced a maximum ethanol concentration of 41.5 ± 0.5 g/L, which was 5% higher (*P* < 0.01) than the obtained by the parental strain (Fig. 3) and represented 50% of the theoretical maximum ethanol that could be obtained in SSF (yield estimated considering the total glucose and xylose that can be potentially available during SSF process and a maximum sugar-to-ethanol conversion yield of 0.51 g/g). The evolved cells also exhibited improved xylose uptake rates, which increased the xylose consumption by about 10% (32% of xylose was consumed after 72 h of SSF). The high xylose:glucose ratio utilized during ALE was decisive for the success of the process since the utilization of xylose as carbon source during the evolution procedure is a key factor to increase the yeast affinity for this sugar. This improved xylose fermenting capacity could be due to improved xylose transport kinetics^38,39^. As a matter of fact, increased expression of hexose transporters was reported in evolved xylose-utilizing yeasts^39–41^, as may be the case for the resulting evolved strain in this study as well.

### Differential gene expression of the improved phenotype

**Figure 4:**
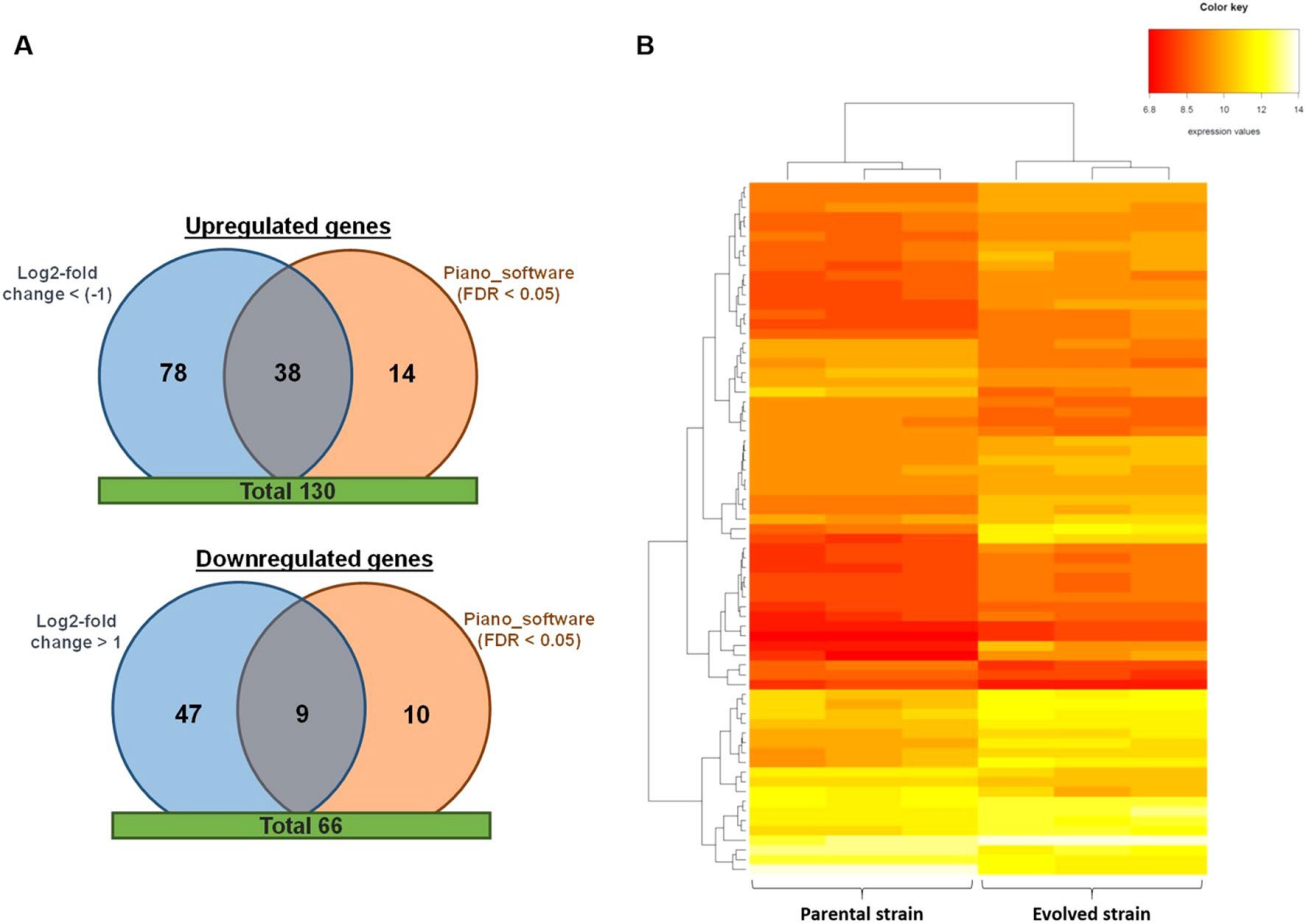
Differential expression analysis between parental and evolved *S. cerevisiae* F12 in terms of (**A**) induced and repressed genes and (**B**) hierarchical clustering. Piano Software [http://biomet-toolbox.chalmers.se].

A total of 196 genes were found upregulated (130 genes) or downregulated (66 genes) in evolved cells in the presence of both solids (20% w/w) and inhibitors (80% v/v of inhibitory mix) (Fig. 4A). These conditions of solids and inhibitors were the most challenging conditions to which cells were evolved in the ALE and thus they were selected for differential gene expression analysis. The differences between parental and evolved cells were also analyzed by hierarchical clustering, which clearly plotted two different groups (Fig. 4B): i) one corresponding to parental cells and ii) another one corresponding to evolved cells. This result supported the differences between *S. cerevisiae* F12 and the corresponding evolved strain.

Differentially expressed genes (parental *vs* evolved) were subsequently analyzed by gene ontology (GO) analysis to determine the biological processes induced and repressed. This analysis highlighted cell cycle (e.g., cytokinesis, regulation of cell cycle, reproductive process) and cell wall organization or biogenesis (e.g., fungal-type cell wall organization, sexual sporulation) as major upregulated biological processes, while maltose metabolic process, transport (e.g., ion transport, amino acid transport, water transport) and homeostatic process (e.g., iron ion homeostasis) were the main biological processes downregulated (Table 4). In spite of identifying several biological processes induced and repressed in the improved phenotype, enrichment analysis identified no metabolic pathway statistically upregulated or downregulated. It is also important to remark that a significant number of identified upregulated (53 genes, ca. 40%) and downregulated (19 genes, ca. 30%) genes had an unknown molecular function (Supplementary Table S1). Furthermore, about 90% of these genes have a Log2-fold change above one order. These results might indicate the potential role of these genes during the cell response to insoluble solids, and therefore, they should be further investigated.

**Table 4:** Upregulated and downregulated biological processes in evolved *S. cerevisiae* F12 cells.

Biological Process Enriched	P-value^a^	Genes	GO term
**Upregulated**			
Cell cycle	1.99E-08	*YBR038W, YBR098W, YDL055C, YDL101C, YDL222C, YER095W, YGL021W, YGL116W, YGR044C, YGR108W, YGR221C, YHR023W, YHR061C, YHR152W, YHR153C, YHR172W, YIL050W, YIL131C, YIL158W, YJR092W, YKL096W, YML027W, YML052W, YML085C, YMR001C, YMR029C, YMR032W, YMR078C, YMR117C, YMR199W, YNL196C, YNR009W, YOL069W, YOL132W, YOR026W, YOR301W, YOR315W, YOR372C, YOR373W, YPL256C, YPL257W, YPR119W*	GO:0,007,049
Cell wall organization or biogenesis	1.70E-06	*YBR038W, YBR067C, YBR076W, YDL055C, YDL222C, YDR261C, YER011W, YHL028W, YHL043W, YHR143W, YIL123W, YJL158C, YKL096W, YKL096W-A, YKL164C, YKL187C, YML052W, YMR215W, YMR305C, YNL283C, YOL030W, YOL132W, YOR247W*	GO:0,071,554
**Downregulated**			
Maltose metabolic process	2.25E-05	*YBR297W, YBR298C, YBR299W, YDL247W, YGR287C*	GO:0,000,023
Transport	6.68E-03	*YAL067C, YBL042C, YBR068C, YBR069C, YBR298C, YDL247W, YEL065W, YER145C, YGR055W, YGR295C, YHL035C, YHL040C, YHL047C, YKL220C, YKR093W, YLL038C, YLL048C, YLL051C, YLL052C, YLL053C, YLR047C, YLR214W, YLR237W, YNL328C, YOR382W, YOR384W, YPL265W*	GO:0,006,810
Homeostatic process	1.65E-02	*YEL065W, YER145C, YHL040C, YHL047C, YKL220C, YLL051C, YLR047C, YLR136C, YLR214W, YOR382W, YOR384W, YPL156C*	GO:0,042,592

^a^Multiple testing was analyzed by Holm-Bonferroni test correction.

**Figure 5:**
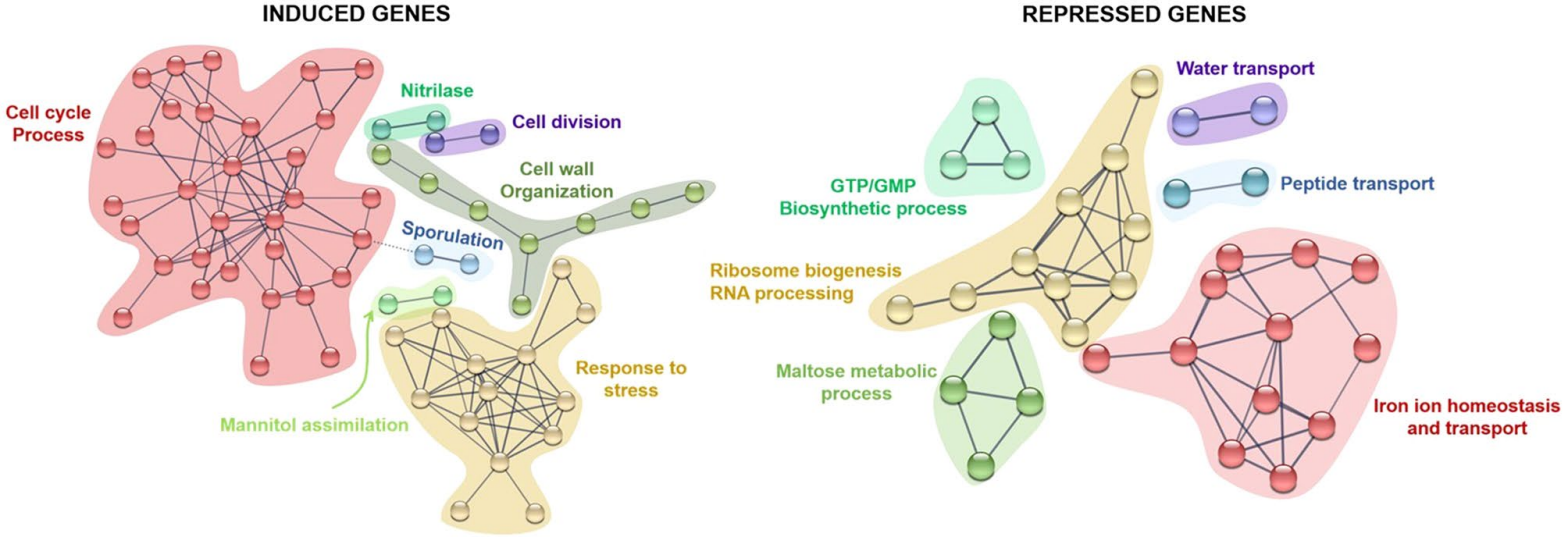
STRING analysis showing protein–protein interactions between induced and repressed genes. STRING software v11 [https://string-db.org/].

The results obtained by GO analysis regarding induced and repressed biological processes were also supported by the protein–protein interaction networks resulting from STRING analysis. STRING revealed cell cycle process, response to stress and cell wall organization as the main upregulated processes, while homeostasis, ribosome biogenesis and transport were highlighted as major downregulated processes (Table 5, Fig. 5). From these analyses, it is important to highlight the upregulation of genes specifically related with DNA damage and the cell response to stress. These genes included for instance *CDC5*, *CTF18*, *HTA1*, *MMS4*, *PLM2*, *RNR1*, *RAD51*, *DUN1*, *SSA3*, *TRR2*, *CTT1*, *ALD3*, *ALD2*, *PAI3*, *SIP18*, and *GRE1*. *CDC5* is known to prevent the cell-cycle arrest induced by the DNA damage checkpoint, allowing cell division and promoting the adaptation of cells to this cell state^42^. Simultaneously, *DUN1*, *CTF18*, *RNR1* and *RAD51* genes were also induced in the evolved *S. cerevisiae* F12 strain. These genes are also related with the DNA damage replication checkpoint and DNA repair mechanisms^43–45^. The overexpression of these genes might prevent cells from having an excess of mutations during cell adaptation, thus encouraging cell survival. The response to stress was also induced through the overexpression of genes involving the protection against oxidative stress (*TRR2*, *CTT1*), heat shock (*SSA3*, *SPG4*) and osmotic stress (*PAI3*), as well as genes related to the general response to stress (*GRE1*, *SIP18*, *ALD2*, *ALD3*). It is worth highlighting that the overexpression of *CTT1* improved xylose utilization in recombinant strains^32^, supporting the overexpression of this gene after ALE that may be responsible of the increased xylose consumption in the evolved *S. cerevisiae* F12.

**Table 5:** STRING analysis of induced and represses genes after evolution of *S. cerevisiae* F12.

Biological process	Genes^a^
**Upregulated**	
Cell cycle process	*BUB3, CDC5, CDC20, CDC21, CHS2, CLB1, CLB2, CLN1, CLN2, CTF18, DUN1, FDO1, FKH1, HHO1, HOF1, HTA2, KIN3, MMS4, MYO1, NDD1, NRM1, NUD1, NUF2, PCL7, PLM2, RAD51, RNR1, SPC24, SPC97, SPO12, TUB1, YOX1*
Response to stress	*ALD2, ALD3, CTT1, FMP45, GRE1, HBT1, HXT5, PAI3, PHM7, SIP18, SPG4, SSA3, TRR2, YEF1*
Cell wall organization	*CIS3, GAS5, GIC1, SCW10, SIM1, SRL1, TOS1, TOS2, (CWP2, WSC4, WSC2)*
Sporulation	*RME1, SGA1*
Cell division	*BUD4, RAX1*
Mannitol assimilation	*DSF1, HXT13*
Nitrilase	*NIT1, YIL165C*
**Downregulated**	
Iron ion homeostasis and transport	*ARN1, ARN2, ENT4, FIT2, FRE1, FRE2, FRE5, FRE6, FRE8, FTR1, SIT1, TIS11*
Ribosome biogenesis, RNA processing	*CMS1, ECM2, FAL1, FCF2, HGH1, NOP14, NOP7, ROK1, YCR016W, YNL050C*
Maltose metabolic process	*IMA1, MAL32, MAL31, MAL33 (MPH2)*
GTP/GMP biosynthetic process	*IMD1, IMD2, IMD3*
Peptide transport	*BAP2, PTR2, (TAT1, MUP1, DIP5)*
Water transport	*AQY2, YLL053C*

^a^Upregulated and downregulated genes with similar functions and highlighted by GO analysis are listed in brackets.

Specific genes (8 in total) related with cell wall organization were also induced (Tables 4 and 5). Among them, *SRL1*, *CWP2*, *WSC2* and *WSC4* encode important proteins for the stabilization of the cell wall^46–48^. The overexpression of these genes might specifically be related with the yeast response against the stress promoted by solids. The presence of insoluble solids during yeast growth promotes the formation of cavities that cause a change in the external morphology of cells from a round-turgid shape to a highly wrinkled morphology^12^. Overexpression of the aforementioned cell wall proteins might counteract this effect and maintain cell wall integrity under the stress conditions.

Major downregulated biological processes include ribosome biogenesis and RNA processing, as well as the transport of specific molecules including iron, peptides and water (Table 5). Repression of protein synthesis is one of the first cell responses upon stress exposure (heat shock, osmotic and oxidative stress), as it is a highly energy consuming process^49,50^. Nevertheless, although having the general protein synthesis process repressed, cells can simultaneously induce the translation of stress-related genes to face the adverse environmental conditions^51^. This was also the case for the evolved *S. cerevisiae* F12 in this work. The second main downregulated biological process was transport. Most of the transport-related genes are associated to peptide/amino acid transport and to iron ion transport and homeostasis (Tables 4 and 5). In this work, repression of peptide/amino acid transport genes might be linked with the downregulation of protein biosynthesis upon stress exposure. On the other hand, it is highly remarkable the relatively high number of genes (up to 12 genes) that are involved in iron ion transport and homeostasis, including the transporter-encoding genes *FIT2*, *FTR1*, *SIT1*, *ARN1* and *ARN2*, and genes encoding different ferric reductases (*FRE1*, *FRE2*, *FRE5*, *FRE6*, *FRE8*). Iron is an essential element required for different biological processes such as respiration, synthesis of nucleic acids, carbon metabolism, as well as photosynthesis and nitrogen fixation^49^. However, iron may be toxic for cells due to its oxidative capacity in the ferrous form, which increases the importance of having a tight control of the iron metabolism. A high intracellular concentration of reactive oxygen species (ROS) under oxidative stress conditions represents a potential threat since the interaction between ROS and iron may end up in the formation of new hydroxyl radicals with increased prooxidant capacity^52^. The simultaneous presence of both insoluble solids and lignocellulose-derived inhibitors during fermentation processes causes a severe oxidative damage in yeast cells, which greatly increases the intracellular ROS levels^12^. This high ROS concentration might be responsible for repressing the corresponding iron-related genes as a way to reduce the risks associated to a marked oxidative stress. Yeast cells (and other multicellular organisms) usually promote iron depletion to prevent metal toxicity and the irreversible damage under oxidative stress conditions^52^.

Overall, these results clearly show the complex inhibitory environment that cells have to face during lignocellulosic biomass conversion. In response to a single stressor, specific genes and pathways have been identified as key components to increase yeast robustness. For instance, *ZWF1* has been identified as a key element during oxidative stress in *S. cerevisiae* upon exposure to a wide variety of chemical and environmental stress agents^53^. During a heat shock, changing ergosterol by fecosterol alters membrane fluidity rendering thermotolerance in yeast^54^. The general response to stress and the cell cycle arrest have been identified as important processes to face a high concentration of insoluble solids^12^. By contrast, in lignocellulose-conversion processes cells must simultaneously deal with a bunch of chemical inhibitors and a high concentration of insoluble solids. To cope with such adverse conditions, this study demonstrate that cells should be capable of maintaining cell membrane integrity and preventing oxidative damage. Therefore, upregulation of membrane-related genes (e.g. *SRL1*, *CWP2*, *WSC2* and *WSC4*) and induction/repression of genes and pathways involving the oxidative stress and the general response to stress (e.g.* CDC5*, *DUN1*, *CTT1*, *GRE1*, *FTR1*, *ARN1*, *FRE1*) can be targeted in future studies to evaluate cell robustness in lignocellulose-related bioprocesses.

## Conclusions

The presence of insoluble solids and lignocellulose-derived inhibitors synergistically increased their inhibitory potential exerted on *S. cerevisiae* F12, especially when using xylose as major carbon source. After subjecting *S. cerevisiae* F12 to an ALE, the resulting evolved cells showed better fermentation performance in terms of higher xylose fermentation efficiency and ethanol yield than the parental strain. Differential gene expression analysis revealed the induction of genes related with cell wall integrity and the response to stress, as well as the repression of protein biosynthesis and the iron transport and homeostasis as main biological processes responsible for the improved phenotype. These results pointed out the necessity of further developing yeast strains less susceptible to the effects caused by all the stress agents present during the conversion of lignocellulosic materials, providing some molecular insights of the mechanism that yeast uses to face these stressors.

## Supplementary Information

Supplementary Table S1.
