# Supplementary material for: Insights into cell robustness against lignocellulosic inhibitors and insoluble solids in bioethanol production processes
Source: Sci Rep. 2022 Jan 11;12:557. doi: 10.1038/s41598-021-04554-4 (PMC8752620; doi:10.1038/s41598-021-04554-4)
Supplement: Supplementary file 1 — Supplementary Table S1. [file 41598_2021_4554_MOESM1_ESM.docx]

**Supplementary Table S1.** Upregulated and downregulated genes found during differential expression analysis between parental and evolved Saccharomyces cerevisiae F12 with unknown biological process

| **Gene name** | **Brief description** | **Log2-fold change** |
| --- | --- | --- |
| **Upregulated**: | | |
| *AIM20* | Protein whose biological role is unknown; localized to nucleus, cytoplasm, and vacuole | 1.6 |
| *AIM34* | Protein whose biological role and cellular location are unknown | 1.3 |
| *ASF2* | Nuclear protein involved in chromatin silencing at silent mating-type cassette | 1.1 |
| *ECM34* | Protein whose biological role is unknown; localizes to the cytosol | 1.0 |
| *ECM8* | Protein whose biological role and cellular location are unknown | 1.4 |
| *ERP5* | Protein with similarity to Emp24p and Erv25p; involved in ER to Golgi transport | 0.6 |
| *FAR8* | Protein involved in recovery from arrest in response to pheromone | 0.8 |
| *FAT3* | Protein required for fatty acid uptake | 2.3 |
| *FDO1* | Protein involved in donor selection during mating type switching; localizes to the nucleus in different large-scale studies | 1.8 |
| *FLO11* | Protein involved in flocculation, coflocculation, cell adhesion during biofilm formation, and in pseudohyphal and invasive growth; localizes to the plasma membrane, the bud neck and the extracellular region | 4.0 |
| *FMP45* | Protein involved in cell wall organization and ascopore formation; localizes to cell periphery, cell cortex, plasma membrane, and mitochondria | 1.4 |
| *GAS3* | Possibly inactive member of the GAS family of GPI-containing proteins; localizes to the cell wall | 1.4 |
| *GAS4* | Protein involved with Gas2p in spore wall assembly; localizes to the cell wall | 1.2 |
| *GAS5* | Protein similar to Gas1p; localizes to the cell wall | 0.5 |
| *GRE1* | Protein whose biological role is unknown; localizes to the cytoplasm in different large-scale studies | 3.7 |
| *HBT1* | Shmoo tip protein; involved in mating projection formation | 1.5 |
| *HUA2* | Protein whose biological role is unknown; localizes to the cytoplasm in a large-scale study | 1.3 |
| *LPX1* | Triglyceride lipase involved in triglyceride catabolism in the peroxisomal matrix | 1.0 |
| *NCW1* | Protein whose biological role is unknown; localizes to the cytoplasm and endoplasmic reticulum in a large-scale study | 0.8 |
| *NDD1* | Transcriptional activator essential for nuclear division; localized to the nucleus | 1.2 |
| *PIN3* | Negative regulator of actin nucleation-promoting factor activity | 1.1 |
| *PLB2* | Phospholipase B (lysophospholipase) involved in lipid metabolism | 1.1 |
| *PLM2* | Putative transcription factor, contains Forkhead Associated domain | 1.2 |
| *PNS1* | Protein whose biological role is unknown; localizes to the plasma membrane in large scale studies | 1.4 |
| *PRM10* | Predicted integral membrane protein whose biological role is unknown | 1.3 |

**Supplementary Table S1.** Cont.

| **Gene name** | **Brief description** | **Log2-fold change** |
| --- | --- | --- |
| **Upregulated**: | | |
| *RAX1* | Protein involved in bud site selection; localizes to vacuole and bud neck | 1.5 |
| *SFG1* | Nuclear protein putative transcription factor; required for growth of superficial pseudohyphae but not for invasive pseudohyphal growth | 1.9 |
| *SLZ1* | Sporulation-specific protein with a leucine zipper motif | 1.0 |
| *SPC24* | Component of the kinetochore-associated Ndc80 complex; involved in chromosome segregation, spindle checkpoint activity, and kinetochore clustering | 1.4 |
| *SPG4* | Protein whose biological role and cellular location are unknown | 2.2 |
| *SPO12* | Protein involved in meiosis and regulation of mitotic exit; localized to the nucleolus | 1.3 |
| *SPO16* | Protein involved in ascospore formation, protein sumolyation, meiotic recombination and synaptonemal complex assembly; localizes to the condensed nuclear chromosome | 1.1 |
| *SRL1* | Mannoprotein required for cell wall stability in the absence of GPI-anchored mannoproteins | 1.4 |
| *SUR7* | Plasma membrane protein, component of eisosomes | 1.4 |
| *TDA6* | Protein whose biological role is unknown; localizes to the cell periphery and vacuole | 1.6 |
| *TOS1* | Protein whose biological role is unknown; localizes to the cell wall; localized to the vacuole in a large-scale study | 1.1 |
| *TOS2* | Protein involved in anchoring Cdc24p to sites of polarized growth; negative regulator of cytokinesis; localizes to the incipient bud site, the bud tip, and the bud neck | 1.2 |
| *WSC2* | Transmembrane signaling receptor involved in Rho protein signal transduction, heat response, and cell wall organization; localizes to cytoplasm, vacuole, site of polarized growth, bud, and shmoo tip | 1.2 |
| *YBR071W* | Protein whose biological role is unknown; localizes to the cytoplasm and bud neck | 1.3 |
| *YET2* | Predicted integral membrane protein whose biological role is unknown; colocalizes with the ribosome in a large-sclae study | 1.1 |
| *YFL067W* | Protein whose biological role and cellular location are unknown | 1.6 |
| *YHR214C-D* | Protein whose biological role and cellular location are unknown | 1.5 |
| *YIL024C* | Protein whose biological role and cellular location are unknown | 1.4 |
| *YIL165C* | Putative protein of unknown function | 1.5 |
| *YJL118W* | Protein whose biological role is unknown; colocalizes with the ribosome in a large scale study | 1.0 |
| *YML119W* | Putative protein of unknown function; non-essential gene | 1.1 |
| *YMR315W-A* | Protein whose biological role and cellular location are unknown | 1.1 |
| *YNL058C* | Protein whose biological role is unknown; localizes to the vacuole in a large-scale study | 1.4 |
| *YNR014W* | Protein whose biological role is unknown; localizes to the cytoplasm | 2.0 |
| *YOR032W-A* | Protein whose biological role is unknown; localizes to the endoplasmic reticulum | 1.1 |

**Supplementary Table S1.** Cont.

| **Gene name** | **Brief description** | **Log2-fold change** |
| --- | --- | --- |
| **Upregulated**: | | |
| *YOR072W-B* | Protein whose biological role and cellular location are unknown | 1.3 |
| *YPL257W* | Predicted integral membrane protein whose biological role is unknown | 1.4 |
| *YSC83* | Protein whose biological role is unknown; localizes to the mitochondrion and mitochondrial outer membrane in large scale studies | 1.0 |
| **Downregulated genes**: | | |
| *CIS1* | Protein whose biological role is unknown; localizes to the mitochondrion in a large-scale study | -1.7 |
| *CMS1* | Protein whose biological role is unknown; localizes to the cytoplasm, 90S preribosome, and nucleus in different large-scale studies | -0.9 |
| *COS6* | Protein predicted to have a role in the multivesicular body sorting pathway; localizes to the endosome; localizes to the vacuole in a large-scale study | -1.3 |
| *DPA10* | Protein whose biological role and cellular location are unknown | -1.4 |
| *ENT4* | Putative clathrin binding protein, that is localized to cortical actin patches; it has a predicted role in actin filament organization and actin patch assembly, and endocytosis | -1.0 |
| *FCF2* | Nucleolar protein involved in the early steps of 35S rRNA processing; essential gene | -0.8 |
| *FIT2* | Mannoprotein that is incorporated into the cell wall via a glycosylphosphatidylinositol (GPI) anchor; involved in the retention of siderophore-iron in the cell wall | -1.2 |
| *HGH1* | Protein whose biological role is unknown; localizes to the nucleus and cytoplasm in different large-scale studies | -1.0 |
| *IMD1* | Nonfunctional protein with homology to IMP dehydrogenase | -2.3 |
| *PRM5* | Predicted integral membrane protein whose biological role is unknown | -1.1 |
| *PRM7* | Pheromone-regulated protein; predicted to have one transmembrane segment | -1.1 |
| *RRT6* | Predicted membrane protein whose biological role is unknown | -1.1 |
| *RSA1* | Protein involved in the assembly of 60S ribosomal subunits; functions in a late nucleoplasmic step of the assembly | -1.1 |
| *SDO1* | Guanyl-nucleotide exchange factor that contributes to mature ribosome assembly; localizes to nucleus and cytoplasm | -0.7 |
| *YAL065C* | Protein whose biological role and cellular location are unknown | -1.0 |
| *YCR016W* | Protein whose biological role is unknown; localizes to the nucleus and nucleolus in a large-scale study | -0.9 |
| *YGL262W* | Protein whose biological role and cellular location are unknown | -1.2 |
| *YKR041W* | Protein whose biological role is unknown; localizes to the mitotic spindle and the nucleus in large-scale studies | -1.8 |
| *YNL050C* | Protein whose biological role and cellular location are unknown | -0.7 |
